# Characterizing gut microbial dysbiosis and exploring the effect of prebiotic fiber supplementation in patients with COPD

**DOI:** 10.1007/s00394-025-03733-7

**Published:** 2025-06-07

**Authors:** Lieke E.J. van Iersel, Rosanne J. H. C. G. Beijers, Sami O. Simons, Lisanne T. Schuurman, Sudarshan A. Shetty, Guus Roeselers, Ardy van Helvoort, Annemie M. W. J. Schols, Harry R. Gosker

**Affiliations:** 1 Department of Respiratory Medicine, NUTRIM Institute of Nutrition and Translational Research in Metabolism, Maastricht University Medical Centre +, P.O. Box 5800, Maastricht, 6202 AZ The Netherlands; 2 Danone Research & Innovation, Utrecht, The Netherlands

**Keywords:** COPD, Gut microbiota, Inflammation, Nutritional supplementation, Prebiotic fibers

## Abstract

**Purpose:**

Chronic Obstructive Pulmonary Disease (COPD) is associated with poor dietary quality that may lead to gut microbiota imbalances. A healthy gut microbiome contributes to metabolic health and immune function through production of metabolites like short-chain fatty acids. Prebiotic fibers positively influence microbiota by promoting the production of beneficial metabolites. This study aimed to assess altered gut microbiota composition in patients with COPD and to explore the effects of targeted multi-nutrient supplementation including prebiotic fibers on these outcomes.

**Methods:**

An exploratory analysis was performed within the double-blinded placebo-controlled NUTRECOVER-trial to gain preliminary insights into the effects of the nutritional intervention. The cross-sectional baseline comparison included 32 patients with COPD and 32 age-matched healthy references. Subsequently, patients were randomly assigned to a multi-nutrient supplement including prebiotic fibers, vitamin D, tryptophan, and N-3 long-chain poly unsaturated fatty acids (*n* = 16) or placebo (*n* = 16) for three months. Stool samples, blood samples and food diaries were obtained before and after the intervention.

**Results:**

Higher relative abundance of *Bacteroidota* (0.50 ± 0.13 vs. 0.41 ± 0.14, *p* = 0.010), and lower *Firmicutes* (0.40 ± 0.14 vs. 0.49 ± 0.12, *p* = 0.007) were found in patients compared with healthy controls. Patients also showed lower alpha diversity (5.80 ± 0.32 vs. 5.99 ± 0.30, *p* = 0.017) and higher inter-individual variability (0.51 ± 0.16 vs. 0.48 ± 0.10, *p* < 0.001). No effects of the nutritional intervention on gut microbiome and systemic inflammation were shown at 3 months.

**Conclusion:**

Patients with COPD exhibit differences in gut microbiota composition compared with healthy controls. Three months of multi-nutrient supplementation is insufficient to show changes in microbiome composition. The ongoing NUTRECOVER-trial will show the potential of long-term prebiotic fiber supplementation in this susceptible patient population.

**Trial registration number:**

clinicaltrials.gov: NCT03807310.

**Supplementary Information:**

The online version contains supplementary material available at 10.1007/s00394-025-03733-7.

## Introduction

Chronic Obstructive Pulmonary Disease (COPD) is a heterogeneous lung condition characterized by persistent, often progressive, airflow limitation and chronic respiratory symptoms such as cough, increased sputum production and shortness of breath [1]. It is well-known that tobacco smoking and air pollution are predominant risk factors for COPD [1]. Additionally, there is also increasing evidence that poor dietary quality increases COPD risk and accelerates disease progression [2, 3]. In Western society, patients with COPD often adhere to a diet, which is low in dietary fibers and high in saturated fats and refined grains which impacts the gut microbiome [4–6].

The gut microbiota is comprised of a diverse community of microorganisms living in the gastrointestinal tract, including bacteria and viruses that play a crucial role in digestion, nutrient absorption, immune functioning and general health [7, 8]. In the colon, *Bacillota* (synonym *Firmicutes*), *Bacteroidota* (synonym *Bacteroidetes*), *Pseudomonadota* (synonym *Proteobaceria*) *and Actinomycetota* (synonym *Actinobacteria*) are the most abundant phyla [9]. These bacterial phyla may influence health status as they are able to produce numerous metabolites during anaerobic fermentation such as propionate, butyrate and acetate, known as short chain fatty acids (SCFAs). SCFAs support gut health by serving as energy source, enhancing gut barrier integrity and modulating immune signaling [10, 11]. Immunomodulatory functions of SCFAs include stimulation of expression of anti-inflammatory cytokines, promotion of regulatory T cell activity and hence inhibiting inflammatory responses [12, 13]. Dietary intake, especially fiber intake, is a key determinant for gut microbiota composition and diversity [11, 14]. The production of SCFAs in the intestinal tract is associated with dietary fiber intake, as these fibers are converted into SCFAs by bacteria [15].

The gastrointestinal and respiratory tract share a mucosal immune system, which is known as the (bi-directional) gut-lung axis [16, 17]. The gut-lung axis facilitates the excretion of microbial metabolites, cytokines and endotoxins into the bloodstream [18]. Disturbances in the microbial composition in the gut contribute to chronic inflammation and are linked to disease progression in the lungs [16, 18]. It is suggested that the gut microbiota composition can be altered in COPD due to common antibiotic treatments during infections and a poor dietary intake. By poor dietary intake, we are specifically referring to insufficient nutrient intake, meaning that patients are not consuming the necessary amounts of essential nutrients required for optimal health. These factors may contribute to dysbiosis in the gut microbiota, which may subsequently result in a weakened immune response to respiratory infections [11, 19, 20]. One study found 146 microbial species between patients with COPD (GOLD I-IV, mean age 67 years) and healthy individuals (mean age 60 years) [21]. Another study reported a *Prevotella*-dominated gut enterotype and lower levels SCFAs, especially in patients with advanced COPD (i.e. GOLD III-IV) compared to healthy controls [22]. However, the evidence on the exact differences in gut microbiota composition between patients with COPD and healthy individuals is still limited. Moreover, these studies have been conducted in non-European regions and did not adequately control for age, which may affect the results on gut microbiota composition [21, 22]. The first aim of this study was to explore differences in the gut microbiota composition of patients with COPD (GOLD II-IV) and confirm enhanced levels of systemic inflammation compared to age-matched healthy controls in the Netherlands. We hypothesize that patients with COPD exhibit an altered gut microbiota composition compared to healthy controls, alongside increased levels of systemic inflammation in blood.

The gut microbiota may be a therapeutic target for nutritional interventions by potential beneficial effects on microbiota composition and systemic inflammation status [23]. Prebiotics selectively promote the growth of beneficial bacteria in the gut. Most data on the impact of prebiotics on the functional potential of the gut microbiota– such as the ability to produce beneficial metabolites like SCFAs and its benefits for the host are derived from short intervention studies (e.g. ≤ 6 months) investigating the fibers fructo-oligosaccharides (FOS), galacto-oligosaccharide (GOS) and inulin, however longer term data (e.g. > 6 months) are lacking [24, 25]. It has been demonstrated that prebiotic fibers like inulin and FOS can influence gut microbiota composition and reduce markers of inflammation [25]. A clinical trial showed increased fecal bifidobacteria after GOS supplementation for 5 consecutive days in healthy adults [26]. When dietary fiber intake is low, as often seen in patients with COPD, supplementation with prebiotics may restore gut dysbiosis and modulate the gut microbiota composition towards a less inflammatory profile [11]. However, to date, no clinical trials with prebiotic supplementation have been conducted yet in patients with COPD. Therefore, the second aim of the current exploratory study is to evaluate the effects of 3-month low caloric, targeted multi-nutrient supplementation compared to placebo on microbiota composition and levels of systemic inflammation in patients with COPD. We hypothesize that prebiotic supplementation will lead to favorable changes in the gut microbiota, which may, in turn, result in reduced inflammatory markers in patients with COPD. This study is a nested study within the NUTRECOVER-trial, in which a multi-nutrient supplement is being investigated that in addition to prebiotic fibers also contains vitamin D, tryptophan, and N-3 long-chain poly unsaturated fatty acids (N-3 LCPUFAs) as main components predominantly based on previous clinical trials in this patient’s population. We aim to provide preliminary insights into the potential beneficial effects of the prebiotic supplement on a disturbed gut microbiome, which is a common issue in patients with COPD.

## Materials and methods

### Subjects and study design

**Fig. 1:**
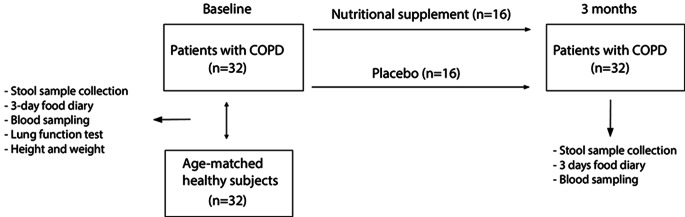
Study design and measurements of the cross-sectional comparison at baseline to compare patients with COPD versus age-matched healthy subjects (*N* = 32 per group) and the randomized controlled trial to compare 3 months of nutritional supplementation to placebo in patients with COPD (*N* = 16 per group)

**Fig. 2:**
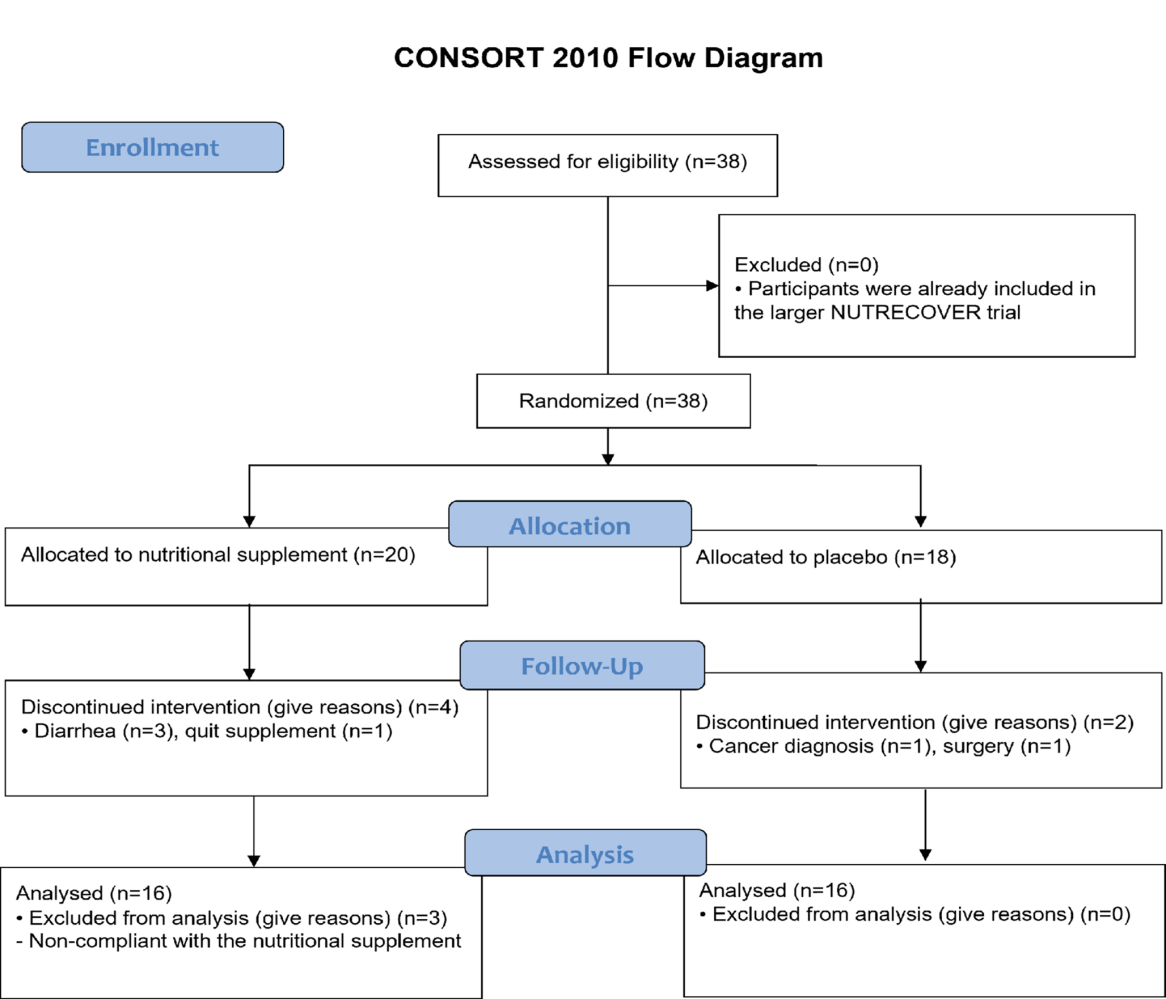
CONSORT flow diagram of the randomized controlled trial

A single center randomized placebo-controlled double-blind clinical trial was conducted in moderate to very severe but clinically stable patients with COPD (Figs. 1 and 2). This exploratory nested study was part of the larger clinical NUTRECOVER-trial at Maastricht University Medical Centre (MUMC+), The Netherlands [27]. Patients with COPD (GOLD II-IV, medically stable [1]) were recruited via their attending lung physicians and via advertisements at the department of respiratory medicine of Maastricht University Medical Centre+ (MUMC+). For baseline comparison of the gut microbiota composition, an age-matched healthy control group was included. These subjects (normal lung function (ratio forced expiratory volume in the first second (FEV_1_) and forced vital capacity (FVC) > 0.7)) were recruited via advertisements between 2021 and 2023 in Maastricht, the Netherlands. The minimization method was used to make sure that the mean age and gender distribution of the subjects in the reference group was comparable to the patients with COPD [28, 29]. This method adjusts participant allocation to reduce imbalances in key characteristics like age and gender. Detailed in- and exclusion criteria of the larger NUTRECOVER-trial are presented in Online Resource 1. The study was approved by the Medical Ethics Committee from MUMC+, Maastricht, The Netherlands (NL66543.068.18/METC18-011), and was conducted in accordance with the principles of the Declaration of Helsinki (Fortaleza, Brazil, 2013) and with the Dutch Regulations on Medical Research Involving Human Subjects Act (WMO). All subjects gave written informed consent before participation. The study has been registered at clinicaltrials.gov (NCT03807310).

### Nutritional supplement

Patients with COPD received the low-caloric active nutritional supplement or an isocaloric isonitrogenous placebo for 3 months consecutively, supplied by Danone Research & Innovation, Utrecht, The Netherlands. Subjects with COPD were randomly assigned to one of the intervention groups by an independent researcher. The study products were provided as flavored powder in sachets and were blinded for both subjects and investigators until the end of the study. The powder needed to be reconstituted in 60 mL water or fruit juice before oral intake and subjects were instructed to consume one sachet per day. The active, low-caloric, supplement provided 6 g prebiotic fibers (20% of RDI): short-chain galacto-oligosaccharides (scGOS), long-chain fructo-oligosaccharides (lcFOS) and low viscosity Pectin (ratio 9:1:2). In addition, the active supplement consists of more components that we hypothesized to be relevant for the other outcomes we will evaluate in the NUTRECOVER-trial (NCT03807310). As per the rationale in Beijers et al. [27] it also contains 1 g N-3 long-chain poly unsaturated fatty acids (N-3 LCPUFAs) (500% of RDI) 600 mg eicosapentaenoic acid (EPA) and 400 mg docosahexaenoic acid (DHA), 200 mg tryptophan (80% of RDI), 20 µg vitamin D (200% of RDI) and supportive micronutrients, containing 94 kCal in total (Online Resource 2) [30]. The placebo product contains the same amount of protein fraction, lipid fraction and digestible carbohydrate, however the amino acids have been replaced by L-alanine and fish oil is replaced by sunflower oil. Contents of minerals and vitamins will not be added to the placebo product. The dose of the nutritional components are all within the recommended safety levels [31]. Supplement and medication intake of the subjects were checked by self-report. Healthy subjects did not receive the nutritional supplement.

### Measurements

#### Blood and stool sample collection and 3-day food diary

Fasting blood samples were collected on each measurement day and serum and plasma was stored at -80 °C until analysis. The gut microbiota composition was measured in stool samples of patients with COPD and healthy subjects. Within 24 h before the measurement day, subjects collected stool samples from one single bowel movement at home in a provided container [22]. The consistency of the stool samples was scored using the Bristol stool form scale [32], ranging from 1 (i.e. hard lumpy) to 7 (i.e. watery/liquid stools). Stool samples (∼ 2 g aliquots) were stored immediately after arrival at -80 °C until processing. Prior to the measurement days subjects were instructed to record all the food and drinks for two weekdays and one weekend day using a 3-day food diary [33]. They were asked to record the time of consumption, the type of food, the brand name and a detailed description of the food, the amount consumed and anything that was added to the food (sugar, salt etc.). Average dietary intake of fibers was reported [34].

#### Lung function

Post-bronchodilator spirometry of all patients and healthy subjects was obtained at baseline. FEV_1_ and FVC were measured by standardized equipment and software using the Vyntus Spiro system (Vyaire Medical, USA), in accordance with the international guidelines [35]. The diagnosis of COPD was considered in an individual with relevant exposure and a post-bronchodilator FEV1/FVC < 0.7 (Gold 2024).

### Sample analysis

#### Systemic inflammation and nutrient levels

Markers of systemic inflammation: high-sensitivity C-reactive protein (hs-CRP), interleukin-6 (IL-6), interleukin-8 (IL-8), interleukin-10 (IL-10), tumor necrosis factor alpha (TNF-α) and interferon gamma (IFN-γ) in blood serum were determined (UMC Utrecht, the Netherlands). For the simultaneous quantification of the cytokines, the Ella instrument (Bio-Techne, Dublin, Ireland) was used. All samples were thawed, vortexed and centrifuged at 10000xg for 5 min at RT. Next, 25 uL of each of the prepared sample was mixed with 25 uL of assay buffer and loaded into the custom made 32 × 6 Ella cartridge. After adding the washing buffer into the cartridge, it was loaded into the Ella instrument and run in accordance with the general Ella instrument protocol using Ella Simple Plex Runner software. After completion of the run, Ella Simple Plex Explorer software was used to process and export the data. The values of the measured cytokines were above the lowest level of quantification (LLOQ). Fatty acid profiles were analyzed in serum samples by gas chromatography as previously described (Danone Research & Innovation, Utrecht, the Netherlands) [36]. The fatty acid concentrations of DHA were monitored for compliance with the nutritional supplement during the intervention phase. In case DHA concentration levels remained unchanged or decreased after three months intervention, subjects were considered non-compliant and were excluded from the analysis.

#### Gut microbiome parameters

To assess metabolic activity of the gut microbiota, physiological parameters were measured in fecal samples: pH, SCFA levels (i.e. acetic acid, propionic acid, (iso)butyric acid and (iso)valeric acid (mg/g)), ammonia (µg/g) and calprotectin (µg/g). Details on the methods have been described previously [26]. Briefly, pH was measured directly in the fecal samples at room temperature using a pH meter (Schott, Mainz, Germany). The SCFAs were quantitatively determined as previously described using the Shimadzu-GC2025 gas chromatograph equipped with a flame ionization detector including hydrogen as mobile phase. For measuring ammonia, samples were fixated with TCA (30% w/v) to a final concentration of 3% TCA per sample and an ammonia (rapid) Assay kit (Cat. Number K-AMIAR Megazyme) was used. Calprotectin levels in the fecal samples were quantified with the Bühlmann Calprotectin ELISA kit (Bühlmann Laboratories AG, Schönenbuch, Switzerland) according to the manufacturer’s protocol.

#### Microbiome community profiling

DNA extraction from the fecal samples (0.2 g) was performed with the QIAmp DNA Stool Mini Kit (Qiagen) following the manufacturer’s protocol, with modifications. The modification consisted of an addition of two bead-beating steps, each consisting of 3 × 30 s rounds on a FastPrep-24 instrument (program 5.5). DNA extracts were stored at − 80 °C until sequencing. The V3-V4 region of the 16 S rRNA gene was PCR-amplified and sequenced on the (MiSeq) platform (Illumina) using the 2 × 300 bp paired-end protocol. Sequencing was performed by Danone Research & Innovation, Singapore. The read pairs were demultiplexed and trimmed (q > 20) using cutadapt v4.1 before being merged using PEAR v0.9.6 [37]. Merged reads with q > 25 over a window of 15 bases, no ambiguous bases and a minimal length of 300 were retained. These were dereplicated and counted using mothur v1.43.0 [38] and unique reads with less than 2 reads over all samples were discarded. Chimeras were removed using VSEARCH v2.13.3 [39], using the ChimeraSlayer reference database [40]. Reads which contained PhiX or adapters as defined in Deblur (part of QIIME v1.9.1) [41, 42] were eliminated. Taxonomic assignment was performed by mothur using the Wang-method (Bayesian classifier) [43] against the SILVA v.138 [44] database. Reads with eukaryotic or chloroplast assignments and those reads that make up less than 1/2000 over all samples were discarded. The sample attributes along with the resulting taxonomic assignment and count table were combined as a TreeSummarizedExperiment v2.6.0 [45] object in R v4.2.1. Further data handling, transformations and calculation of alpha diversity indices was done using the R package mia v1.9.2 [46]. The microbiome data was filtered for abundance at 0.1% and sequences classified as Cyanobacteria and unclassified at Phylum level. Alpha diversity was assessed using the Shannon Index.

### Statistical analyses

Sample size for the cross-sectional baseline comparison was based on expert opinion and statistical power considerations in clinical gut microbiome studies [47]. A total sample size of 50 subjects should be sufficient to pick up differences in microbiota composition between two groups. Based on this, 25 patients with COPD and 25 healthy subjects in the reference group were included for the cross-sectional baseline comparison. Taking a dropout rate of approximately 20% into account, 32 patients with COPD and 32 healthy subjects were included. Subsequently, 16 patients with COPD received the active nutritional supplement and 16 patients received placebo.

Statistical analyses were performed blinded using Graphpad Prism version 8.0.2. Baseline levels were compared using Student t-test in case of continuous variables, *Х*^2^-test for categorical variables and the Mann-Whitney test in case of continuous data that were not normally distributed. Data between groups were analyzed using a two-way repeated measures ANOVA with treatment (nutritional supplement and placebo) and measurement day (pre and post) as within subject factors. In case of significant effects, a *post hoc* analysis was performed using Bonferroni corrections. Correlations between microbiota profile outcomes and blood inflammation markers were assessed, as well as between fecal characteristics and COPD severity using Pearson’s correlation for normally distributed data and Spearman’s rank correlation when the data was not normally distributed. To explore patterns of variation in the microbiota profiles of patients with COPD and healthy controls, a Principal Coordinates Analysis (PCoA) was performed to visualize dissimilarities. A *p* value < 0.05 was considered statistically significant. Data are presented as mean ± standard deviation (SD) when normally distributed and as median (interquartile range) if not normally distributed.

## Results

### Subject characteristics– COPD vs. control

Baseline characteristics are summarized in Table 1. Both healthy controls and patients with COPD were comparable in age, gender and body mass index (BMI) and intake of dietary fibers. Only, as expected, a significant difference in lung function was observed between the groups (Table 1).

**Table 1:** Baseline characteristics

	Unit	Control group (*n* = 32)	COPD patients (*n* = 32)	*p*-value
Age	Years	68.2 ± 8.0	69.8 ± 6.5	0.369
Gender % m/f	% m/f	56/44	69/31	0.439
BMI	kg/m^2^	26.9 ± 5.3	25.0 ± 4.7	0.073
FEV_1_	%predicted	120.9 ± 18.9	54.9 ± 17.5	< 0.001
Dietary fiber intake	g	22.3 ± 8.1	22.3 ± 7.8	0.995

Data are presented as mean ± SD or %. BMI = body mass index; FEV1 = forced expiratory volume in 1 s

### Microbiota profiling and SCFA concentration– COPD vs. control

No significant differences were observed on the metabolic parameters of the fecal samples between the healthy controls and patients with COPD, except for a significantly lower concentration of the SCFAs isobutyric and isovaleric acid in patients with COPD (Table 2). No significant correlations were observed between the characteristics of fecal samples and COPD severity.

**Table 2:** Baseline characteristics of fecal samples

	Unit	Control group (*n* = 32)	COPD patients (*n* = 32)	*p*-value
Ammonia	µg/g	32.0 (24.3, 44.5)	27.0 (23.5, 33.5)	0.103
Calprotectin	µg/g	88.8 (32.8, 187.2)	97.9 (32.1, 320.5)	0.705
pH		6.7 ± 0.6	6.7 ± 0.7	0.948
Acetic acid	mg/g	46.4 (31.5, 57.5)	40.9 (28.8, 65.3)	0.803
Propionic acid,	mg/g	12.6 (9.4, 18.8)	11.4 (7.8, 18.6)	0.373
Butyric acid	mg/g	11.9 (6.4, 20.7)	9.3 (6.4, 15.3)	0.412
Isobutyric acid	mg/g	2.4 (1.7, 2.8)	1.8 (1.2, 2.3)	**0.040**
Valeric acid	mg/g	2.2 (1.4, 2.8)	2.0 (1.4, 2.5)	0.403
Isovaleric acid	mg/g	3.7 (2.7, 4.2)	2.7 (1.7, 3.5)	**0.018**
Total SCFAs	mg/g	76.4 (52.5, 102.9)	71.8 (54.8, 116.8)	0.946
Bristol stool chart		4.0 (4.0, 5.8)	4.0 (4.0, 6.0)	0.303

Data are presented as mean ± SD or median (interquartile range). SCFAs = short chain fatty acids

**Fig. 3:**
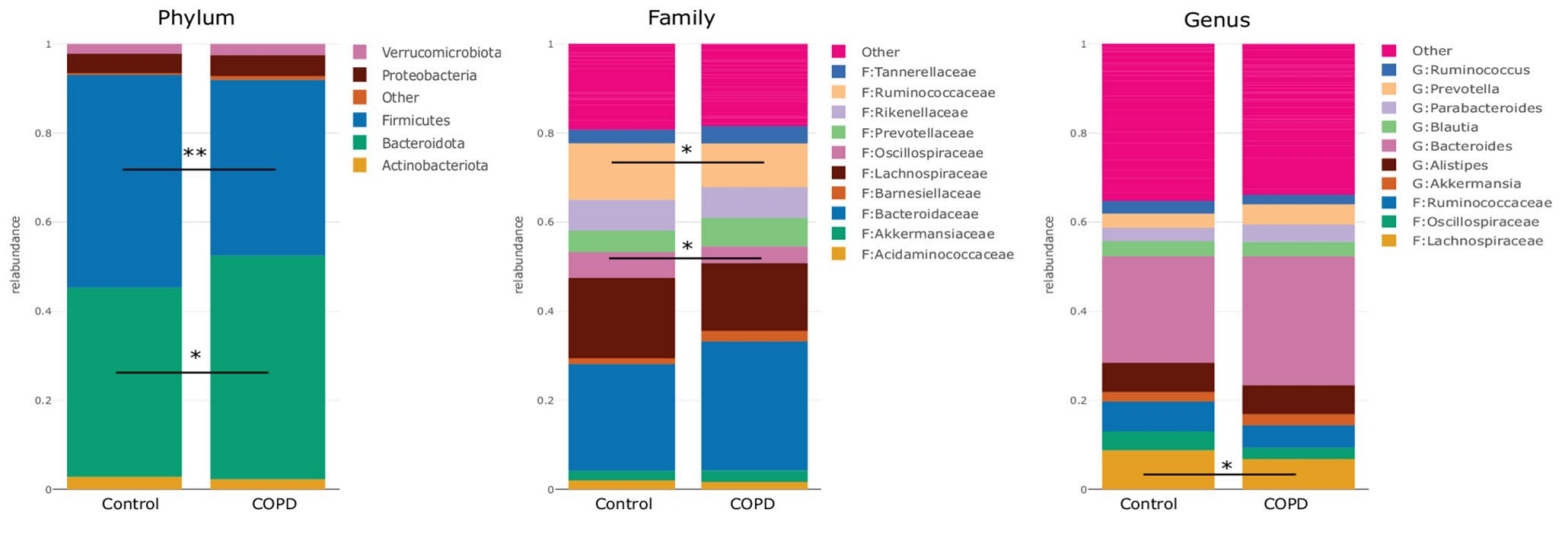
Relative abundances of taxonomic levels (phylum, family and genus) compared with healthy controls (*N* = 32 per group). ^*^p < 0.05; ^**^p < 0.01

**Fig. 4:**
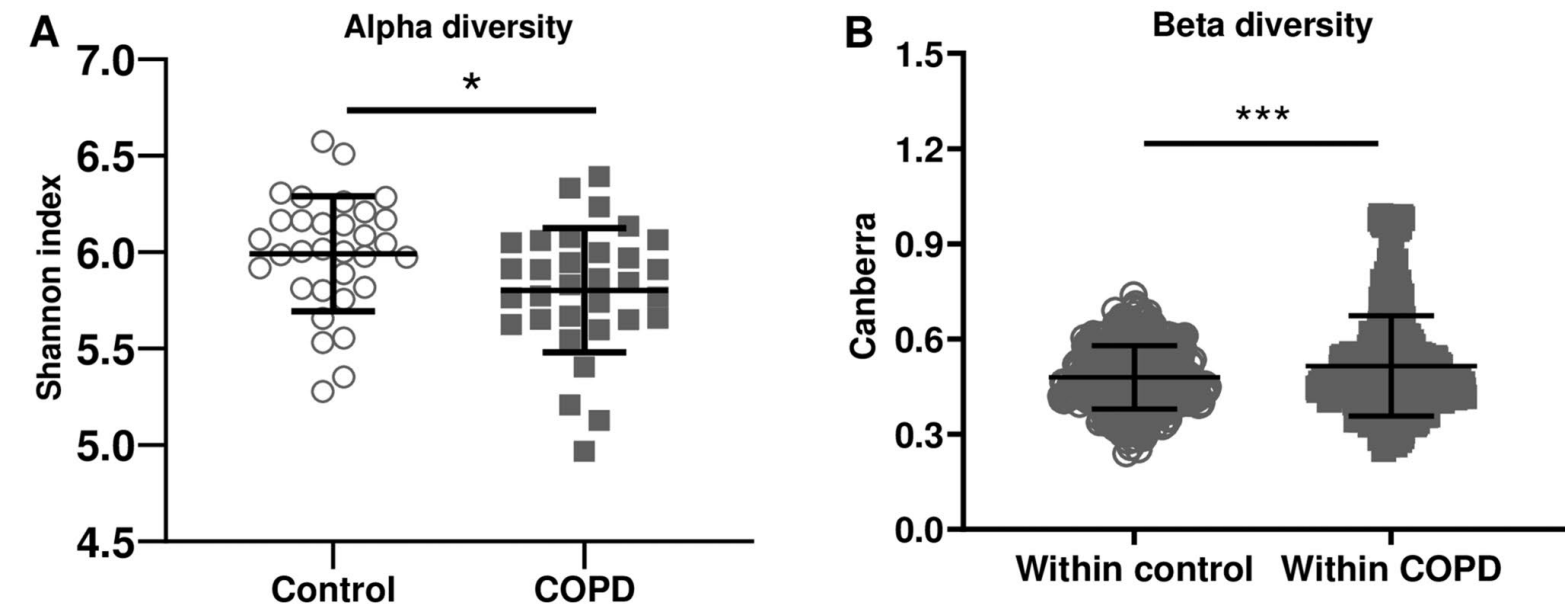
(**A**) Alpha diversity measured by Shannon index compared with healthy controls (**B**) Inter-individual variability in microbiota within patients with COPD compared with controls (*N* = 32 per group). Data are presented as mean ± SD. ^*^p < 0.05; ^***^p < 0.001

**Fig. 5:**
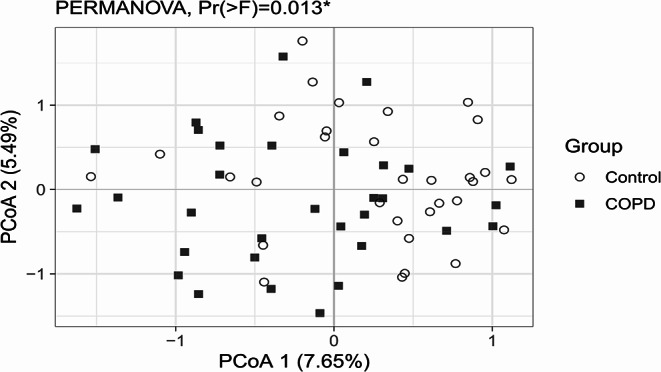
Principal Coordinate Analysis depicting differences in composition and structure of microbiota of patients with COPD and healthy controls (*N* = 32 per group)

Taxonomic compositions were evaluated at phylum, family and genus level (Fig. 3). Cross-sectional comparison between healthy controls and patients with COPD revealed that patients with COPD had a significantly higher relative abundance of *Bacteroidota* (*p =* 0.010*)* and lower relative abundance of *Firmicutes* (*p =* 0.007) at phylum level. Within the *Firmicutes* phylum, the *Rumincoccaceae (p* = 0.029*)* and *Oscillospiraceae* (*p* = 0.027) bacteria at family level were significantly lower in patients with COPD compared with controls. At genus level, only *Lachnospiraceae* were significantly lower in patients with COPD (Fig. 3; *p =* 0.022). Furthermore, patients with COPD exhibited significantly lower alpha diversity in their gut microbiota compared to healthy age-matched controls, as measured by the Shannon index (Fig. 4A; *p* = 0.017). While the alpha diversity was lower in patients with COPD, the inter-individual variability (beta-diversity) in the COPD group was significantly higher compared to controls (Fig. 4B; *p* < 0.001). A Principal Coordinates Analysis (PCoA) plot of microbiota data revealed clustering patterns (Fig. 5). In this plot, microbiota of patients with COPD predominantly clustered in one region, while microbiota of healthy age-matched controls formed a distinct cluster with 7.65% of the variance explained by the first principal component and 5.49% by the second principal component. These components represent the axes of greatest variation in the data, and the clustering observed in the plot reflects the distribution of the microbiota data points in this multidimensional space.

### Nutrient levels and systemic inflammation– COPD vs. control

**Fig. 6:**
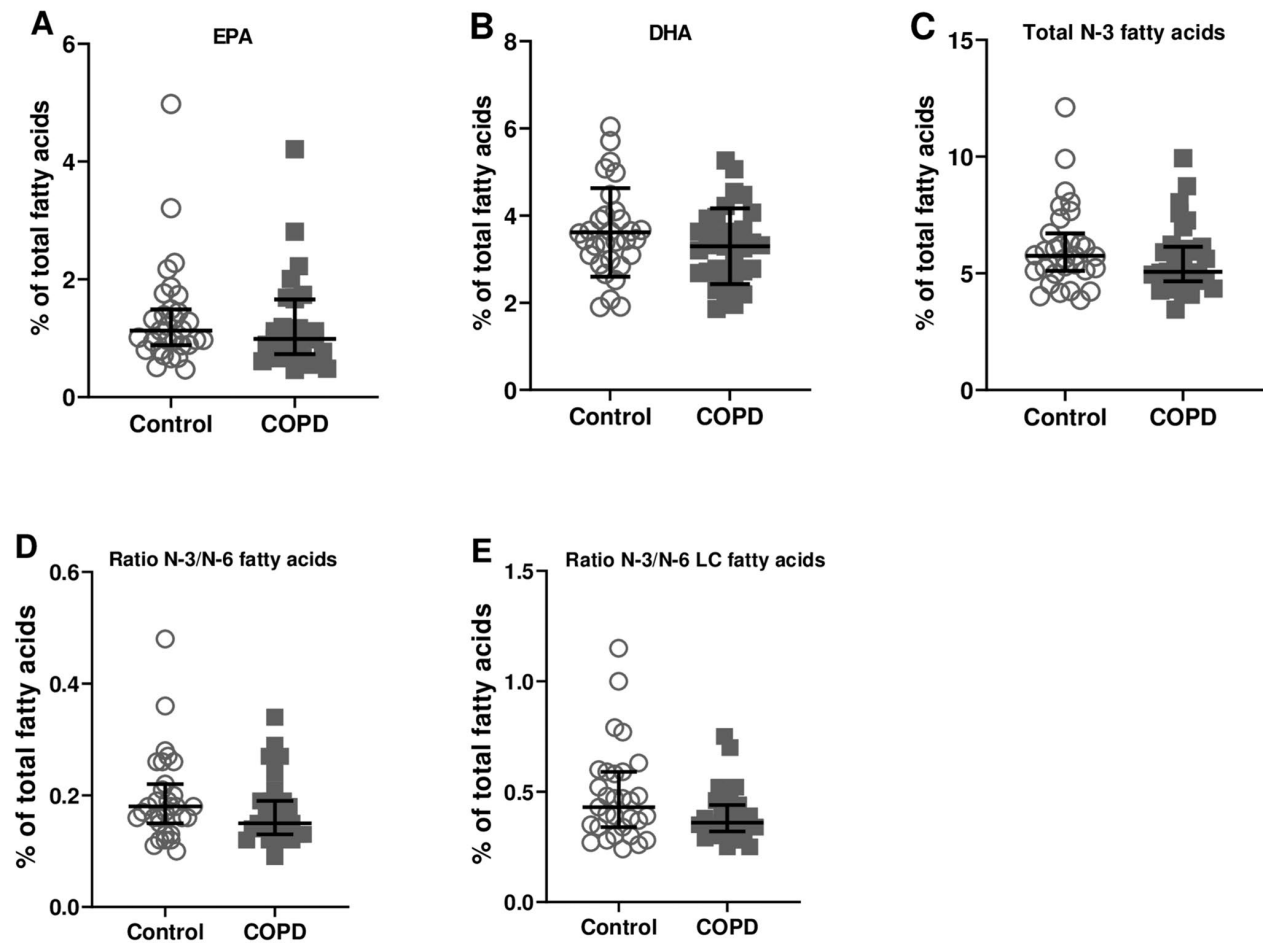
Baseline serum nutrient levels of N-3 PUFAs as relative % of total fatty acids compared with healthy controls. (**A**) EPA (**B**) DHA (**C**) Total level of N-3 fatty acids (**D**) Ratio N-3/N-6 fatty acids and (**E**) Ratio N-3/N-6 long chain fatty acids. *N* = 32 per group. EPA = eicosapentaenoic acid; DHA = docosahexaenoic acid; LC = long-chain. Data are presented as median (interquartile range)

**Fig. 7:**
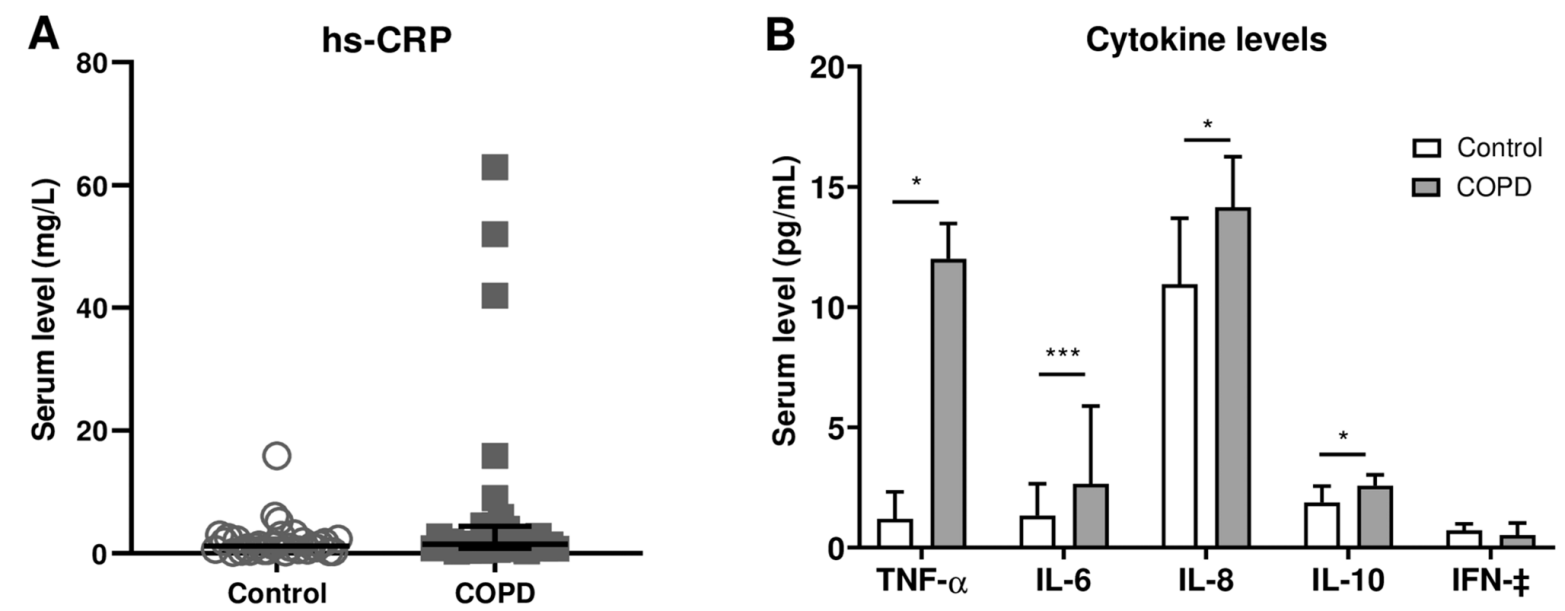
Baseline serum levels of inflammation markers compared with healthy controls. (**A**) High-sensitivity CRP (**B**) Cytokine levels of TNF-α, IL-6, IL-8, IL-10 and IFN-γ. *N* = 32 per group. Data are presented as median (interquartile range). ^*^p < 0.05; ^***^p < 0.001

Although the relative percentages of EPA, DHA, total N-3 fatty acids, ratio N-3/N-6 fatty acids and ratio N-3/N-6 LC fatty acids were numerically lower in patients with COPD compared to controls, they were not significantly different (Fig. 6A-E). Baseline serum level of hs-CRP was comparable between patients with COPD and healthy controls (Fig. 7A). Serum levels of the cytokines TNF-α (*p =* 0.022), IL-6 (*p <* 0.001*)*, IL-8 (*p =* 0.037) and IL-10 (*p =* 0.034) were significantly enhanced in patients with COPD compared with controls (Fig. 7B). No significant correlations were found between systemic inflammation markers and microbiota profiles.

### Subject characteristics and compliance– pre-post intervention (sub-group analysis)

In the nested exploratory part of the larger NUTRECOVER-trial, sixteen patients with COPD were randomly allocated to the placebo group and sixteen to the nutritional supplement group for three consecutive months intervention. All participants completed the study. No significant differences were found at baseline in age, gender, BMI or lung function between the placebo and active intervention group. The amount of dietary fiber intake at baseline was also comparable between placebo and the nutritional supplement group (Table 3). During the 3-month follow-up period, five patients in the placebo group and three patients in the nutritional supplement group received antibiotic treatment. One patient in the nutritional supplement group received four antibiotic treatments within three months.

**Table 3:** Baseline characteristics of patients with COPD in the placebo and nutritional supplement group

	Unit	Placebo (*n* = 16)	Nutritional suppl. (*n* = 16)	*p*-value
Age	years	70.4 ± 5.8	69.3 ± 7.4	0.653
Gender	% m/f	75/25	63/37	0.704
BMI	kg/m^2^	23.8 ± 3.4	26.3 ± 5.5	0.136
FEV_1_	%predicted	51.6 ± 16.8	58.1 ± 18.0	0.300
Dietary fiber intake	g	23.5 ± 6.3	21.1 ± 8.9	0.379

Data are presented as mean ± SD or n. BMI = body mass index; FEV1 = forced expiratory volume in 1 s

**Fig. 8:**
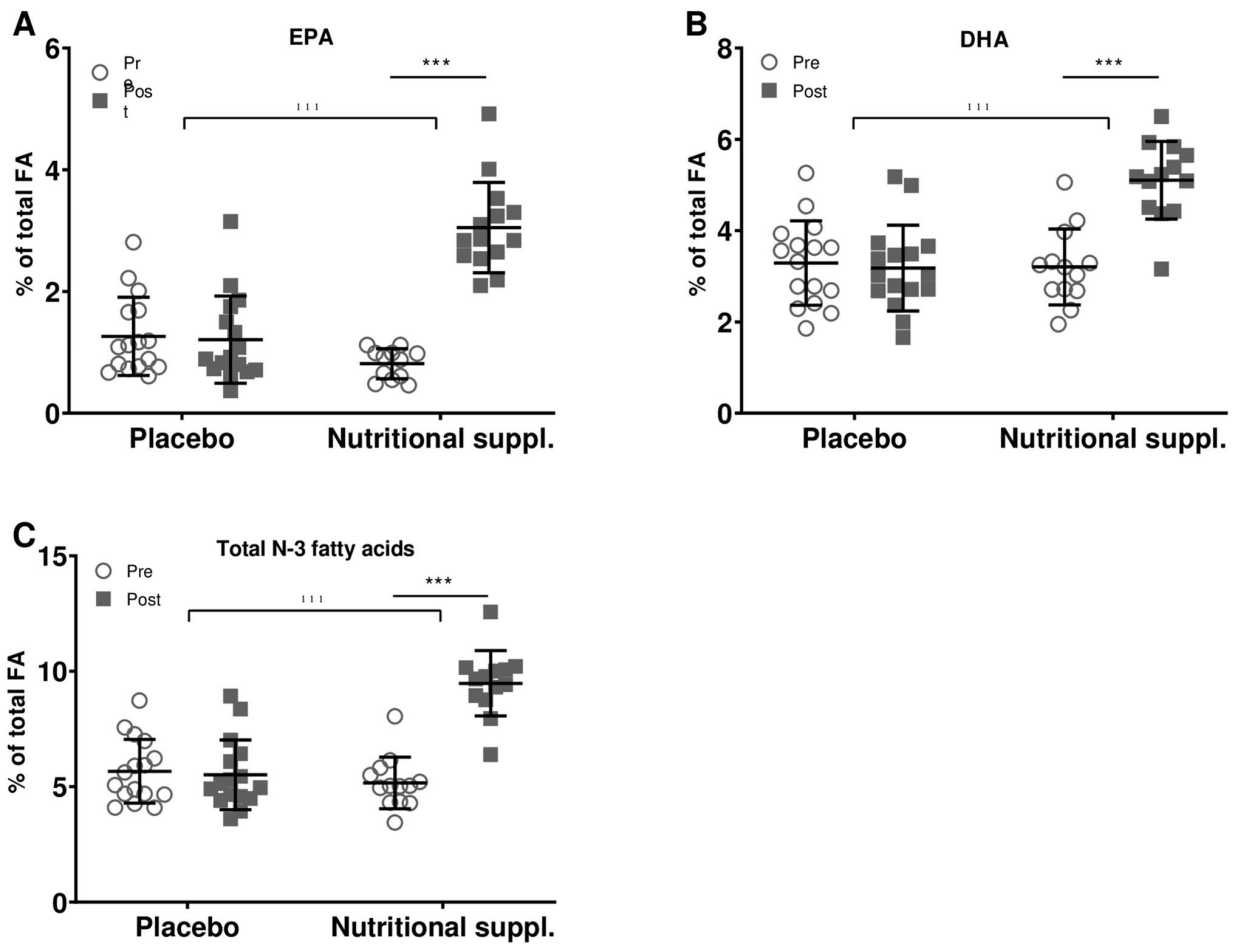
Serum nutrient levels of N-3 fatty acids as relative % of total fatty acids pre-post intervention. (**A**) EPA (**B**) DHA (**C**) Total level of N-3 fatty acids. *N* = 16 for placebo and *n* = 13 for nutritional supplement. EPA = eicosapentaenoic acid; DHA = docosahexaenoic acid. Data are presented as mean ± SD. ^***^p < 0.001

The fatty acid profiles were evaluated for the pre-post intervention as a compliance marker, showing a significant interaction effect (*p* < 0.001) for EPA, DHA and total N-3 fatty acid concentrations. Relative % of EPA, DHA and N-3 fatty acids of total fatty acids were significantly increased by the nutritional supplement compared to placebo (*p* < 0.001, Fig. 8A-C). In total, three of the sixteen patients in the nutritional supplement group did not seem compliant as their DHA and EPA serum concentration remained similar or even decreased after three months supplementation: therefore, they were excluded from the analyses.

### Microbiota profiling and SCFA concentration– pre-post intervention

In this small exploratory study, the administration of the prebiotic fiber supplement over a period of 3 months did not yield any statistically significant effects on the metabolic parameters of the fecal samples in comparison to placebo (Table 4). Moreover, there were no statistically significant alterations observed either within the groups or between them in terms of beta-diversity or taxonomic composition (data not shown).

**Table 4:** Characteristics of fecal samples and dietary fiber intake of patients with COPD in the placebo and nutritional supplement group

	Unit	Placebo (*n* = 16)		Nutritional suppl. (*n* = 13)		*p*-value
		Pre	Post	Pre	Post	
Ammonia	µg/g	27.0 (24.0, 36.8)	23.0 (15.8, 38.3)	28.0 (21.3, 33.8)	26.5 (22.0, 37.3)	0.403
Calprotectin	µg/g	63.1 (22.1, 252.3)	114.9 (24.7, 503.3)	128.5 (48.9, 343)	226.4 (91.0, 374.4)	0.742
pH		6.7 ± 0.7	6.8 ± 0.8	6.7 ± 0.7	6.8 ± 0.6	0.736
Acetic acid	mg/g	40.5 (28.1, 76.0)	47.8 (30.6, 58.9)	41.1 (31.2, 58.7)	39.4 (32.0, 69.3)	0.562
Propionic acid	mg/g	11.8 (7.1, 19.4)	13.6 (10.3, 16.6)	11.2 (7.8, 17.2)	15.0 (8.9, 23.3)	0.059
Butyric acid	mg/g	10.6 (5.2, 15.2)	12.9 (5.4, 19.1)	8.1 (6.4, 16.3)	9.4 (7.3, 20.0)	0.594
Isobutyric acid	mg/g	2.0 ± 1.1	1.9 ± 0.8	1.9 ± 0.8	2.0 ± 0.5	0.624
Valeric acid	mg/g	2.4 ± 1.2	2.0 ± 0.8	1.8 ± 0.7	2.1 ± 0.9	0.056
Isovaleric acid	mg/g	3.0 ± 2.0	2.6 ± 1.3	2.7 ± 1.4	2.8 ± 0.8	0.526
Bristol stool chart		4.0 (4.0, 5.8)	5.0 (4.0, 6.0)	4.0 (3.0, 6.0)	4.0 (4.0, 6.0)	0.859
Alpha diversity	Shannon index	5.8 ± 0.4	5.7 ± 0.5	5.8 ± 0.3	5.4 ± 0.5	0.235
Dietary fiber intake*	g	23.2 (18.3, 27.2)	19.6 (16.5, 24.9)	19.9 (15.5, 23.0)	14.6 (11.5, 21.3)	0.880

Data are presented as mean ± SD or median (interquartile range). *Dietary fiber intake was determined based on the 3-day food diary and does not include 6 g of prebiotic fibers provided by the supplement

### Nutrient levels and systemic inflammation– pre-post intervention

**Fig. 9:**
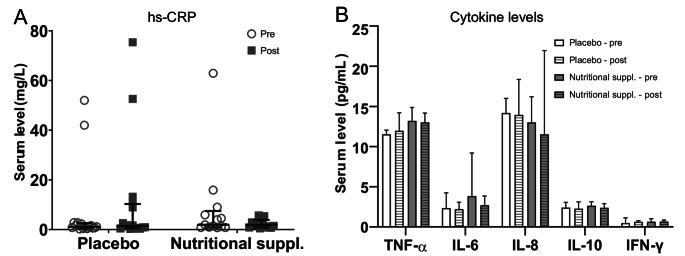
Serum levels of inflammation markers pre-post intervention. (**A**) High sensitivity CRP (**B**) Cytokines TNF-α, IL-6, IL-8, IL-10 and IFN-γ. *N* = 16 for placebo and *n* = 13 for nutritional supplement. Data are presented as median (interquartile range)

No significant effect by the nutritional supplement was observed on systemic inflammation markers, including hs-CRP and serum levels of cytokines TNF-α, IL-6, IL-8, IL-10 and IFN-γ (Fig. 9).

## Discussion

In the current study, we first compared the gut microbiota composition and markers of systemic inflammation of patients with COPD to age-matched healthy references. Secondly, the effects of three-month prebiotic supplementation on gut microbiota composition and markers of systemic inflammation were investigated in patients with COPD. To our knowledge, this is the first clinical study exploring the effects of prebiotic supplementation in this patient population. We observed distinct differences in the gut microbiome composition of patients with COPD compared to healthy controls. Patients exhibited a higher relative abundance of *Bacteroidota* and a lower relative abundance of *Firmicutes* on phylum level, along with reduced levels of *Rumincoccaceae* and *Oscillospiraceae* on family level. Patients with COPD also demonstrated lower alpha diversity and higher inter-individual variability. As expected, elevated levels of inflammatory cytokines were detected in patients with COPD compared to healthy age-matched controls. However, the nutritional intervention did not yield significant effects on microbiota composition and structure after 3 months.

The microbial change in patients with COPD compared to controls that was observed in this study is consistent with prior findings and may be attributed to a diet high in fat and animal protein, which may indicate a common microbial signature associated with COPD [22, 48, 49]. However, contrary findings have also been reported, where results pointed towards an increase in the *Firmicutes* phylum and lower amount of *Bacteroidetes* within the gut microbiota of patients with COPD and those suffering from exacerbations, defined as acute worsening of respiratory symptoms that results in additional therapy, compared to controls [50, 51]. They suggested that this altered *Firmicutes*-to-*Bacteroidetes* ratio may be a potential biomarker for acute exacerbations of COPD [51]. The relative abundance of these phyla seems highly variable in the same population, which is likely attributable to various ethnic and lifestyle-related factors like physical activity level and smoking status that influence the composition of the gut microbiota [52]. *Bifidobacteria* were not observed among the top 10 most abundant bacteria in the current study. The relative abundance of *Bifidobacteria* was consistently low among all participants. This absence might be influenced by the age of the participants, as *Bifidobacteria* levels naturally decline with age [53]. Age-related changes in the gut microbiota can also affect microbial responses to prebiotic interventions, suggesting that age-specific strategies might be necessary to optimize the effects of such interventions, particularly in older populations [54]. A lower concentration of SCFAs isobutyric and isovaleric acid in fecal samples of patients with COPD compared to controls was observed, which aligns with previous research in patients with COPD and in a particulate matter-induced rat model of COPD. Decreased levels of SCFAs, including isobutyric acid and isovaleric acid, may indicate dysbiosis in the gut microbiota of patients with COPD as they are produced by beneficial bacteria [22, 55, 56]. We also found that the gut microbiota of patients with COPD was characterized by higher inter-individual variability together with lower microbial diversity compared to healthy controls. These findings are consistent with the results reported in Li et al., but not with Yan et al. [22, 51]. The higher inter-variability may be a result of the heterogeneity of the COPD population [57].

In the intervention phase of this small exploratory study, the prebiotic supplementation did not yield significant changes in the gut microbiota composition, on (anti-)inflammatory cytokine levels or diversity measures in patients with COPD compared to placebo after three months. One factor to consider is the potential impact of the heterogeneity of the COPD population on interventions. Variations in disease severity, medication use, stress levels, presence of comorbidities, and lifestyle factors such as dietary patterns, physical activity level, body composition and smoking status can influence the microbiota composition [58, 59]. Technical limitations related to the sampling and/or the responsiveness to prebiotic supplementation may have influenced the microbiota outcomes. There was a delay of up to 24 h between fecal collection and freezing, and some metabolites may have been lost during sampling or measurement. Additionally, SCFAs produced from fermentable fibers may have been absorbed in the proximal part of the large intestine before reaching the feces, making detection more difficult. Another factor is habitual dietary fiber intake, which is suggested to be generally low in patients with COPD [5]. In the current study, patients with COPD reported a dietary fiber intake of 22 g per day, which is similar to that of healthy controls and is not considered as low or deficient [60, 61]. This adequate fiber intake may explain why no significant effect of the prebiotic supplement on the gut microbiota composition was observed. It is important to note that this is based on self-reported intake, which may be subject to misreporting [62]. One problem with the self-reported dietary fiber intake is that the type of fiber consumed by the patients was not specified in their food diaries, making it difficult to determine the specific health effects of the dietary fibers. Due to their fermentability, specific prebiotic fibers included in the supplement may exert distinct health effects compared to dietary fibers derived from, for example, bread sources. Six age-matched healthy references that participated in the study were partners from patients with COPD, meaning that they probably consumed a similar diet, including comparable dietary fiber intake. Intake of prebiotics can lead to varying effects on microbiota composition and SCFA concentration between individuals. The gut microbiota may have a limited ability to produce SCFAs from fibers. In individuals with a chronically low intake of dietary fiber, the gut microbiota may be less adapted to ferment fibers efficiently, potentially limiting SCFA production in response to prebiotic supplementation. Therefore, prebiotic strategies need to be tailored to account for baseline fiber intake, ensuring that the dose and type of prebiotic are sufficient to induce a beneficial effect [63, 64]. Furthermore, long-term dietary patterns are predominantly associated with significant alterations in specific gut enterotype patterns, while short-term interventions typically do not result in sustained or significant changes in these patterns [65, 66]. To achieve long-term effects on gut microbiota individuals need to consistently increase their intake of fiber-rich foods such as whole grains, fruits, and vegetables, supported by a healthy lifestyle [67]. Long-term studies (e.g. >6 months), with approaches tailored to their unique microbial and inflammatory profiles, may provide more insights into optimizing prebiotic use for sustained gut microbiota benefits in COPD. It is also possible that we missed the initial change in microbiota that potentially occurred in the first few days due to only having a single time point at three months.

The current nested exploratory study has several strengths and limitations. Strengths of this study include its controlled design, where both a COPD population and age-matched control group were included. Additionally, the nutritional intervention seems to be practical and manageable, as shown by levels of compliance among participants. The smaller sample size of the intervention phase is a limiting factor in this exploratory study. However, future results of the NUTRECOVER-trial, in which 166 patients with COPD receive nutritional supplementation for 12 months, may give additional insight into the effects of prebiotic supplementation on a larger scale [27]. In the NUTRECOVER-trial, a multi-nutrient supplement that targets multiple organs simultaneously is being investigated. The nutritional supplement contains not only 6 g of prebiotic fibers, but also other nutrients that can have anti-inflammatory and immune-modulating properties. Specifically, N-3 LCPUFAs could have influenced the results. Growing evidence suggests a correlation between N-3 LCPUFAs and the gut microbiota [68]. They may positively influence the composition of the gut microbial community, while the gut microbiota, in turn, can impact the metabolism and absorption of these fatty acids. However, no effects on microbiota composition were found in the current study. Understanding of the connections between N-3 LCPUFAs and the gut microbiota remains limited and warrants further research [69]. To determine the specific effects of prebiotic fibers, nutritional interventions should include prebiotic fibers without other nutrients.

In conclusion, the current study demonstrates significant dysbiosis and elevated systemic inflammatory markers in patients with COPD, with no significant improvements observed following three-month supplementation including prebiotic fibers. The heterogeneity of the COPD population, sample size, prebiotic fiber dose, potential dietary deficiencies, and the duration of the intervention are important factors to consider in future studies that are elucidating the nutritional therapeutic potential of prebiotics in COPD. Further understanding of the mechanisms underlying the gut-lung axis interactions in COPD may provide further insight in order to develop targeted microbiome-based therapies.

## Electronic supplementary material

Below is the link to the electronic supplementary material.

Supplementary Material 1

## Data Availability

The datasets used and/or analyzed during the current study are available from the corresponding author on reasonable request.
