# Supplementary material for: Characterizing gut microbial dysbiosis and exploring the effect of prebiotic fiber supplementation in patients with COPD
Source: Eur J Nutr. 2025 Jun 7;64(5):210. doi: 10.1007/s00394-025-03733-7 (PMC12145321; doi:10.1007/s00394-025-03733-7)
Supplement: Supplementary file 1 — Supplementary Material 1 [file 394_2025_3733_MOESM1_ESM.docx]

Supplementary information European journal of Nutrition

**Characterizing gut microbial dysbiosis and exploring the effect of prebiotic fiber supplementation in patients with COPD**

Lieke E.J. van Iersel^1^, Rosanne J.H.C.G. Beijers^1^, Sami O. Simons^1^, Lisanne T. Schuurman^1^, Sudarshan A. Shetty^2^, Guus Roeselers^2^, Ardy van Helvoort^1,2^, Annemie M.W.J. Schols^1^, Harry R. Gosker^1^

^1^Department of Respiratory Medicine, NUTRIM Institute of Nutrition and Translational Research in Metabolism, Maastricht University Medical Centre, Maastricht, The Netherlands

^2^Danone Research & Innovation, Utrecht, The Netherlands

Corresponding author: L.E.J. van Iersel

NUTRIM Institute of Nutrition and Translational Research in Metabolism

Maastricht University Medical Centre +, Department of Respiratory Medicine

P.O. Box 5800, 6202 AZ Maastricht, The Netherlands

Tel: +31 88 388 7331

Email: [l.vaniersel@maastrichtuniversity.nl](mailto:l.vaniersel@maastrichtuniversity.nl)

**Online Resource 1** In-and exclusion criteria of patients with COPD and healthy subjects

| **Eligibility criteria** | |
| --- | --- |
| **Inclusion criteria patients with COPD** | - Moderate to very severe COPD according to GOLD criteria (i.e. GOLD stage II-IV) [1]; - Medically stable (no hospital admission <4 weeks prior to the start of the study and no temporary oral steroid or antibiotics use due to a COPD exacerbation in the last 4 weeks). - No allergy or intolerance to components of the study product. |
| **Inclusion criteria healthy subjects** | - Lung function: FEV_1_/FVC ≥ 0.7; - Medically stable. |
|  |  |
| **Exclusion criteria** | - Age <18 years; |
|  | - Other acute or unstable chronic diseases that will compromise the study outcome (e.g. active cancer requiring treatments); |
|  | - Participation in any other study involving investigational or marketed products concomitantly or within four weeks prior to entry into the study; |
|  | - Terminal illness; |
|  | - Lung malignance in the previous 5 years; |
|  | - Diagnosis of dementia or neurodegenerative disease (e.g. Alzheimer’s disease, Parkinson’s disease, Huntington’s chorea, front temporal dementia) in the medical records; |
|  | - Recent diagnosis of cerebral conditions (<1 year e.g. cerebral infarction, hemorrhage, brain tumors, transient ischemic attack) in the medical records; |
|  | - Any medical condition that significantly interferes with digestion and/or gastro-intestinal (GI) function (e.g. short bowel syndrome, inflammatory bowel disease, gastric ulcers, gastritis, (gastro)-enteritis, GI cancer) as judged by the investigator. |

FEV_1_= Forced expiratory volume in 1 s; FVC= Forced vital capacity

**Online Resource 2** Product information nutritional supplement

| **Active Product** | | |  |  | Amounts per serving | | |  | | * |  |  |  |
| --- | --- | --- | --- | --- | --- | --- | --- | --- | --- | --- | --- | --- | --- |
|  |  |  |  |  |  | |  | |  |  |  |  |  |
| **Macronutrients** | |  |  |  | **Micronutrients** | | |  | |  |  |  |  |
|  |  |  |  |  |  |  | |  | |  |  |  |  |
| **Protein fraction** | | **3.6** | g |  | **Vitamins** |  | |  | |  | **Minerals** |  |  |
| L-arginine | | 1 | g |  | Vitamin A | 200 | | ug | |  | Calcium | 200 | mg |
| L-serine | | 1 | g |  | Vitamin B1 | 1.0 | | mg | |  | Phosphorous | 60 | mg |
| L-tryptophan | | 0.2 | g |  | Vitamin B2 | 1.0 | | mg | |  | Magnesium | 65.3 | mg |
| L-aspartate | | 0.5 | g |  | Nicotinamide | 16 | | mg | |  | Zinc | 12 | mg |
| L-cysteine | | 0.2 | g |  | Folate | 277 | | ug | |  | Sodium | 55 | mg |
|  | |  |  |  | Vitamin B6 | 3 | | mg | |  | Potassium | 200 | mg |
| **Lipid fraction** | |  |  |  | Vitamin B12 | 5 | | ug | |  | Chloride | 2.3 | mg |
| Fish oil PUFAs: EPA+DHA | | 1 | g |  | Vitamin C | 157 | | mg | |  | Iron | 10 | mg |
|  | | | | | Pantohenic acid | 6 | | mg | |  | Copper | 0.5 | ug |
|  | | | | | Biotin | 30 | | ug | |  | Manganese | 0.3 | mg |
| **Fiber** | |  |  |  | Vitamin D | 20 | | ug | |  | Molybdenum | 50 | ug |
| Galacto-oligosacharides | | 4 | g |  | Vitamin E | 20 | | mgTE | |  | Selenium | 46 | ug |
| Insulin | | 0.7 | g |  | Vitamin K | 80 | | ug | |  | Chromium | 25 | ug |
| Low viscosity Pectin | | 1.3 | g |  | Nicotinic acid | 4 | | mg | |  | Iodide | 60 | ug |
|  | | | | | **Miscellaneous** | | |  | |  |  | | |
| **Carbohydrates** | | **8** | **g** |  | Choline | 200 | | mg | |  |  | | |
|  | | | | | Taurine | 60 | | mg | |  |  | | |
|  | | | | | Inositol | 15 | | mg | |  |  | | |

*The placebo product contains the same amount of protein fraction, lipid fraction and digestible carbohydrate, however the amino acids have been replaced by L-alanine and fish oil is replaced by sunflower oil. Contents of minerals and vitamins will not be added to the placebo product.*
